# De(C1P)hering the role of ceramide-1-phosphate levels in skin wound healing

**DOI:** 10.1016/j.jlr.2022.100231

**Published:** 2022-05-17

**Authors:** Rashi Agrawal, Wendy B. Bollag

**Affiliations:** 1 Department of Physiology, Medical College of Georgia at Augusta University, Augusta, GA, USA; 2 Charlie Norwood VA Medical Center, Augusta, GA, USA; 3 Department of Dermatology, Medical College of Georgia at Augusta University, Augusta, GA, USA; 4 Department of Medicine, Medical College of Georgia at Augusta University, Augusta, GA, USA

**Keywords:** C1P, ceramide-1-phosphate, CERK, ceramide kinase, HETE, hydroxyeicosatetraenoic acid, S1P, sphingosine-1-phosphate, SphK, sphingosine kinase

The skin is the largest organ of the body and serves several important roles: preventing water loss, serving as the first barrier against trauma—including UV radiation and chemicals—and pathogens, participating in metabolic functions such as vitamin D synthesis and temperature regulation, and informing the body of external conditions through billions of sensory and proprioceptor nerve cells. It is a dynamic organ composed of various cell types that have specific and unique functions, which are present in different skin layers, called the epidermis, dermis, and hypodermis. For example, the outermost layer of the skin, the epidermis, contains melanocytes, which primarily produce melanin to protect against damaging UV radiation, as well as keratinocytes that form a mechanical barrier to the environment; the dermis in the middle contains sweat glands and blood vessels important for body temperature regulation; and the adipocytes of the hypodermis serve in part to insulate the organ systems inside.

These important functions of the skin demonstrate how damage to any part of the skin is concerning and why the body’s response to a wound is so significant. Wound healing is a process of four phases that are regulated by many biological agents that have their own specific timing of induction and function/purpose; these phases are hemostasis, inflammation, proliferation, and tissue remodeling. The physiological events that occur throughout this process are vascular constriction, platelet activation, white blood cell infiltration, re-epithelization, collagen deposition, and vascular maturation (1).

Improper wound healing is quickly becoming a significant burden in US health care because of the aging population, increased rates of comorbidities such as diabetes and obesity, and antibiotic-resistant bacterial infections. Acute and chronic wounds that impact the population range from surgical incisions to pressure ulcers to acute traumatic wounds, with complications arising from infection, malnutrition, and/or lack of patient education. Impaired wound healing can also result from certain comorbidities such as diabetes or hypertension (2). Wound treatment has a dramatic impact on health care expenditures in the United States, with acute wounds resulting in 17.2 million hospital visits (in 2014)—including outpatient and inpatient surgical visits—and Medicare spending estimates totaling from $28.1 billion to $96.8 million for all wound types (also in 2014) (3, 4). As a result, research has become focused on the processes that govern normal wound healing and the factors that contribute to normal versus impaired wound healing.

There has been an interest in understanding the biological markers and signaling molecules that mediate wound healing in order to better develop pharmacological therapies that promote targeted healing. In this issue of the *Journal of Lipid Research*, Maus *et al.* (5) demonstrated that a decrease in the levels of ceramide-1-phosphate (C1P), accomplished either through inhibiting or genetically ablating the anabolic enzyme ceramide kinase (CERK), resulted in a significant acceleration of skin wound healing. In particular, this study used a newly formulated CERK inhibitor, SYR382141, that could be used in mice in vivo and was found to block the production of CERK-derived C1P in a similar manner to a previously established CERK inhibitor. Appropriate dosing of SYR382141 in an acute excisional wound model resulted in a significant increase in wound closure rate over a period of 10 days. It should be noted that these investigators used a splinted wound model, in which the skin wound is prevented from contracting such that the wound heals primarily by re-epithelialization. This provision was important in terms of the possibility of translating the results to humans, since human skin wounds heal primarily by re-epithelialization rather than by contraction, as does rodent skin (2). The mechanism by which CERK inhibition with SYR382141 accelerated wound healing seemed to be due at least in part to the observed increases in the expression of fibroblast activation protein, the number of infiltrating cells, and type 1 collagen deposition (5). These results supported the idea that inhibition of CERK-derived C1P formation may lead to a transition from the inflammatory stage to the proliferation phase required for proper wound healing.

In this study, Maus *et al.* (5) not only examined closure rates of acute wounds in mice but also performed lipidomic and migration analyses after mechanical trauma on isolated mouse dermal fibroblasts that were treated with exogenous CERK inhibitors or were derived from *Cerk*-null mice. Compared with sham controls, wild-type primary dermal fibroblasts treated with a CERK inhibitor or lacking *Cerk* were found to exhibit an increase in migration velocity. In addition, the lipidomic analysis of C1P-reduced primary dermal fibroblasts showed an increase in the lipoxygenase-derived hydroxyeicosatetraenoic acid (HETE) 5-HETE, as well as the biologically active metabolite of 5-HETE, 5-oxo-HETE, compared with the control; this increase was also observed in acute skin wounds with CERK inhibition (5). Dermal fibroblasts from mice genetically engineered to possess a gene encoding a mutant cPLA_2_α (mutant cPLA_2_α knock-in mice) that did not interact with C1P showed similarly enhanced 5-oxo-HETE levels and accelerated migration. Therefore, the authors concluded that CERK-derived C1P is a negative regulator of the production of bioactive 5-oxo-HETE and dermal fibroblast migration at least in part through cPLA_2_α. Further experimentation also showed that 5-HETE/5-oxo-HETE signaling through the G protein–coupled receptor OXER1 enhanced dermal fibroblast migration and promoted wound healing.

The findings from Maus *et al.* (5) are an important contribution to the current body of research on molecular mediators, specifically bioactive lipids, in the important and all-encompassing biological process of inflammation. A study by Pettus *et al.* (6) confirmed that CERK-derived C1P is required for the release of arachidonic acid and production of eicosanoids—both of which are essential for the inflammatory stage of wound healing. The authors supported this relationship by showing that C1P is a direct activator of cPLA_2_α through interaction with the CaLB/C2 domain in response to inflammatory agonists (e.g., cytokines). Blocking the interaction of C1P with cPLA_2_α using the mutant (knock-in) cPLA_2_α or inhibiting CERK-mediated C1P synthesis also results in a decrease in the production of some inflammatory eicosanoids, such as prostaglandin E_2_ (7, 8). Thus, Maus *et al.* (5) combined this finding with the knowledge that C1P concentration is high during the inflammatory stage but decreases later in the healing process (7) to conclude that C1P is a proinflammatory mediator and essential checkpoint for wound maturation.

In addition, a study by Wijesinghe *et al.* (7) employed similar methods as the current study to analyze the lipidomic and migration profiles of fibroblasts after scratch-induced mechanical trauma; in particular, their experiment investigated the specific role that CERK-derived C1P plays in wound healing by creating *Cerk*-null genetically modified mice. The results of this study in *Cerk* knockout mice support the current findings from Maus *et al.* (5)—which primarily focused on a novel CERK inhibitor SYR382141 to inhibit the C1P response by confirming the role of C1P in arachidonic acid release and eicosanoid synthesis. They found that *Cerk*-null mice have a deficient eicosanoid response to mechanical injury and are unable to initiate proper wound healing (7); this result emphasizes the importance of C1P to the inflammatory stage. Concomitantly, the researchers found that *Cerk*-null mice also have abnormal fibroblast migration patterns, which leads to improper tissue remodeling. Finally, Wijesinghe *et al.* (7) further investigated C1P in acute human wounds, where they discovered that the lipid levels increase throughout the inflammatory phase of wound healing and peak during the proliferative phase. This time course supported the overall conclusion that C1P is a biologically significant player in wound healing involved in the progression through the stages of this process.

On the other hand, sphingolipids in addition to C1P may also be involved in regulating wound healing and could partially underlie some effects of CERK inhibition. Thus, C1P is formed by the action of CERK on ceramide. Ceramide can also be deacylated by ceramidases to sphingosine. Whether sphingosine affects wound healing is unclear, but sphingosine can be phosphorylated to sphingosine-1-phosphate (S1P) by sphingosine kinase (SphK). Accumulating evidence supports an important role for S1P in skin wound healing (e.g., (9)). There are two isoforms, SphK1 and SphK2, that produce S1P endogenously. A recent study showed that SphK1 overexpression in wounds via application of a nanoparticle-packaged plasmid promotes skin wound healing in the mouse-splinted wound model (10). This treatment resulted in increased angiogenesis and inflammatory cell recruitment, suggesting these effects as a potential mechanism of action. Topical administration of this nanoparticle led to less scar formation as well (10). Therefore, it seems possible that another mechanism by which CERK inhibition or knockout might promote wound healing is by inducing the accumulation of ceramide with subsequent shunting of its metabolism through ceramidase and SphK to S1P to promote wound healing (Fig. 1).

**Fig. 1:**
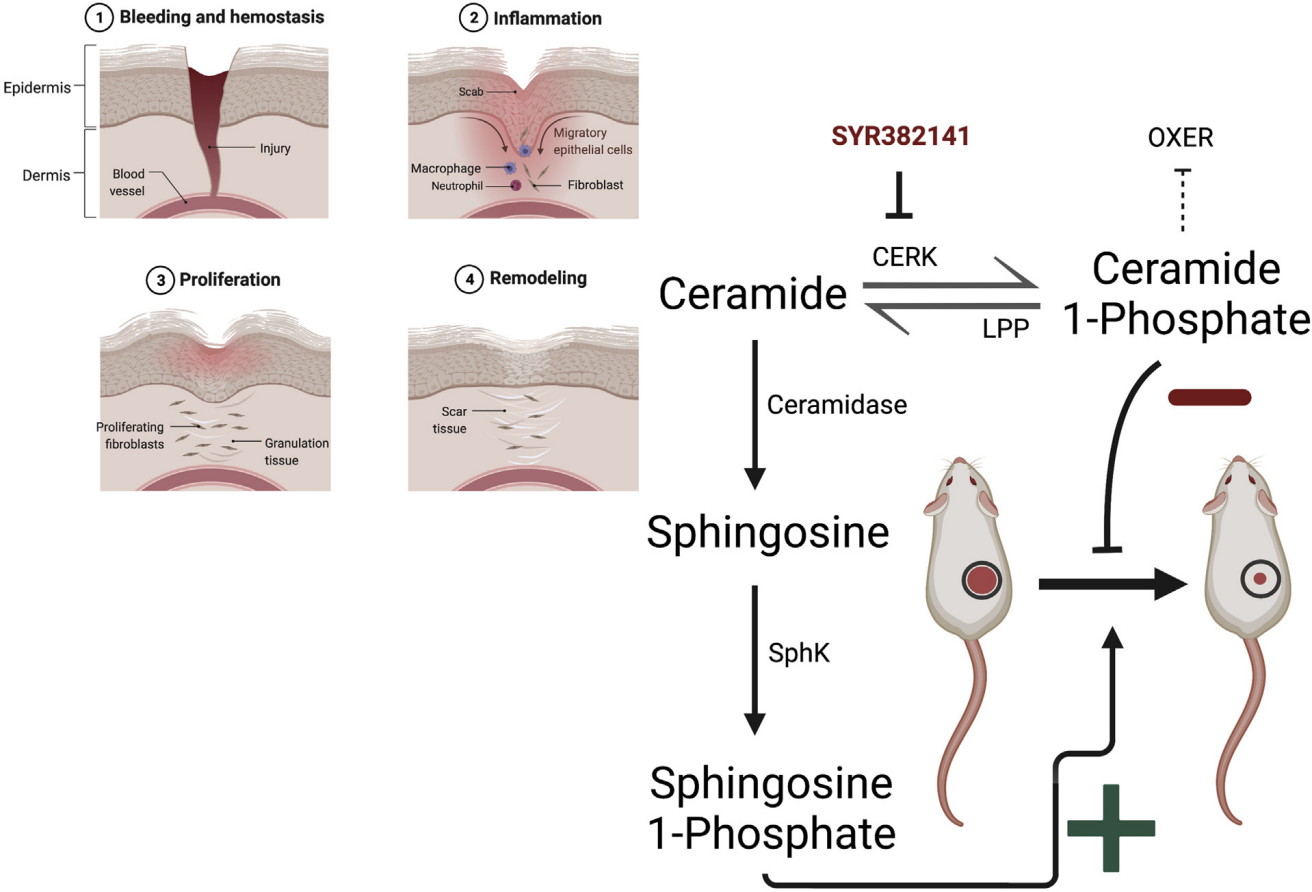
Production of ceramide metabolites and their effects on skin wound healing. Illustrated are the four phases of skin wound healing as well as the metabolism of ceramide, by CERK to C1P and by ceramidases to sphingosine. Sphingosine can then be phosphorylated by SphK to S1P, which seems to promote wound healing. C1P, which has been shown to inhibit wound healing, can be dephosphorylated to ceramide by lipid phosphate phosphatase (LPP). C1P inhibits production of 5-HETE and 5-oxo-HETE, which promote wound healing through the OXER receptor. SYR382141 is the novel CERK inhibitor used in the current study by Maus *et al.* (5). This figure was created with BioRender.com.

In conclusion, the study by Maus *et al.* (5), as well as supporting the literature from the Chalfant laboratory, demonstrated the possibility of CERK-derived C1P being a manipulatable bioactive molecule to promote wound healing. By combining the idea that reduction of C1P levels is essential to the transition past the inflammatory stage of wound healing in animal models and that CERK inhibitors have been successfully used to alter wound healing, there is an opportunity to develop pharmacologic therapies that can help accelerate healing of acute wounds or even reactivate fibroblast proliferation and tissue healing in chronic wounds.

## Conflict of interest

The authors declare that they have no conflicts of interest with the contents of this article. The contents of this article do not represent the views of the Department of Veterans Affairs or the United States Government.
